# Mortality‐to‐incidence ratio of early‐onset colorectal cancer in high‐income Asian and Middle Eastern countries: A systemic analysis of the Global Burden of Diseases Study 2019

**DOI:** 10.1002/cam4.6631

**Published:** 2023-10-20

**Authors:** Majed Ramadan, Rawiah A. Alsiary, Doaa A. Aboalola

**Affiliations:** ^1^ Population Health Research Section, King Abdullah International Medical Research Center (KAIMRC) King Saud Bin Abdulaziz University for Health Sciences, Ministry of National Guard – Health Affairs Jeddah Kingdom of Saudi Arabia; ^2^ Department of Cellular Therapy and Cancer Research, King Abdullah International Medical Research Center (KAIMRC) King Saud Bin Abdulaziz University for Health Sciences, Ministry of National Guard – Health Affairs Jeddah Kingdom of Saudi Arabia

**Keywords:** Asian countries, disparities, early‐onset colorectal cancer (CRC), gender, high‐income

## Abstract

**Background:**

The incidence of early‐onset colorectal cancer (EO‐CRC) has been consistently rising leading to a significant cancer burden among younger adults in Asian and Middle Eastern high‐income countries. The study aims to investigate the survival outcomes of EO‐CRC among high‐income Asian and Middle Eastern populations from 1990 to 2019 using the mortality‐to‐incidence ratio, with a focus on examining the differences in gender.

**Methods:**

This is a systematic analysis of the Global Burden of Disease (GBD) 2019 study. We include individuals aged 15 to 49 years old in high‐income Asian and the Middle Eastern countries. The colorectal cancer mortality‐to‐incidence ratio (MIR) was calculated for both genders by dividing the age‐specific mortality rate per 100,000 for colorectal cancer by the age‐specific incidence rate per 100,000 for each nation in the sample for a given year.

**Results:**

An overall decline in male and female MIR was observed from 1990 to 2019 in Asian and Middle Eastern countries. Ten out of thirteen Asian and Middle Eastern countries had a higher female MIR compared to their male counterparts. The global male MIR was found to be significantly higher than that of female (*p*‐value 0.008, coefficient estimate: 1.51). In Middle Eastern countries, Saudi Arabia had a significantly higher female MIR compared to their male counterparts (*p* < 0.0001, coefficient estimate: 12.65).

**Conclusion:**

This research addresses the knowledge gap concerning gender‐based differences in EO‐CRC survival outcomes in high‐income Asian and Middle Eastern countries, providing insights into the factors influencing these disparities in these regions. Policymakers should focus on developing targeted prevention and treatment programs for women, and addressing cultural and social barriers that may prevent women from seeking timely medical care.

## 1 INTRODUCTION

The incidence of early‐onset colorectal cancer (EO‐CRC), diagnosed before the age of 50, has been consistently rising since the 1980s, resulting in a significant cancer burden among younger adults.^1^ EO‐CRC accounts for approximately 10%–12% of newly diagnosed cases of colorectal cancer.^2^, ^3^ However, the underlying reasons for this upward trend remain largely unclear^2^, ^3^, ^4^ necessitating further research to identify the drivers. Recent studies have highlighted a surge in early‐onset CRC in high‐income Asian countries, including Japan, South Korea, and Taiwan.^5^, ^6^, ^7^, ^8^ In fact, East Asia exhibits the highest incidence of EO‐CRC, with the region experiencing the most rapid increase in incidence rates from 1990 to 2019.^8^ Taiwan, in particular, reports the highest incidence and mortality rates for early‐onset CRC globally.^8^ This pattern extends beyond East Asia, as high‐income countries in the Middle East, such as Saudi Arabia, also observe high EO‐CRC incidence rates.^9^ Despite the alarming incidence rates of early‐onset colorectal cancer in high‐risk populations of Asia and the Middle East, research on this topic remains limited. Therefore, prioritizing EO‐CRC studies in these regions is crucial for gaining a comprehensive understanding and implementing effective disease management strategies.

Colorectal cancer demonstrates significant biological heterogeneity, which is evident in the varying incidence rates of different cancer subtypes across different demographics, including gender.^10^ Gender is a well‐established prognostic factor that is associated with outcomes and survival in CRC.^11^, ^12^, ^13^, ^14^ While gender is widely recognized as a determinant of CRC incidence, there have been limited investigations into gender differences in outcomes for EO‐CRC and whether these differences vary over time.^15^ Furthermore, although population‐based studies have identified determinants of CRC incidence in various populations, there is currently no existing research specifically examining gender differences in EO‐CRC survival across Asian and Middle Eastern countries.^16^, ^17^, ^18^ Therefore, it is crucial to explore gender‐specific differences in EO‐CRC outcomes within high‐risk populations to identify effective strategies for preventing and managing CRC in young individuals.

In this study, we utilized the mortality‐to‐incidence ratio (MIR) as a novel measure to assess cancer mortality in relation to incidence, which serves as a proxy for 1‐survival.^19^, ^20^ MIR has gained popularity and reliability among researchers.^21^, ^22^, ^23^ This measure allows for the identification of countries where mortality rates exceed what would be expected based on their incidence rates. The MIR has been established as a valuable tool for evaluating cancer registries and assessing the quality of cancer care and reporting within a single‐country context, as demonstrated by Parkin and Bray.^24^ They found that the MIR, which is approximately equal to 1 minus cancer survival, can provide insights into the completeness of a cancer registry.^24^, ^25^ Furthermore, this measure has been employed to examine cancer disparities between different ethnic groups, such as African Americans versus European Americans in South Carolina, as demonstrated by Hebert et al.^26^, ^27^ Therefore, this current study aims to investigate the survival outcomes of EO‐CRC among high‐income Asian and Middle Eastern populations from 1990 to 2019 by utilizing the mortality‐to‐incidence ratio, with a specific focus on comprehensively examining the differences in gender.

## 2 METHOD

### 2.1 Data acquisition

The data were obtained from The Global Burden of Disease (GBD) 2019. The GBD provided inclusive and accessible epidemiological data on 369 diseases and injuries, as well as 87 risk factors, from 1990 to 2019. These comprehensive data encompass 7 super‐regions, 21 regions, and more than 200 countries and territories, and were obtained using a rigorous methodology previously described.^28^, ^29^

### 2.2 Definition of Early‐Onset Colorectal Cancer (EO‐CRC)

In this study, we classified all cancers identified as C18‐21, D01.0‐D01.2, and D12‐D12.9 according to the 11th revision of the International Classification of Diseases as CRCs. We included both colon and rectal carcinomas based on the colorectal continuum model.^28^ EO‐CRC was defined as CRC diagnosed before the age of 50; particularly, we used the age category of 15 to 49 years old. To obtain the necessary data, we utilized the Global Health Data Exchange (GHDx) tool (https://ghdx.healthdata.org/gbd‐2019, accessed on March 15, 2023) and selected “colon and rectum cancer” as the “cause” and “incidence,” and “deaths,” as the “measure.” In addition, then we followed the inclusion criteria for selecting the included countries and regions.

### 2.3 Inclusion and exclusion criteria

This study focused on EO‐CRC population aged 15 to 49 years old in high‐income countries located in Central Asia, Southeast Asia, East Asia, and the Middle East. The Asian countries included were China, Japan, the Republic of Korea, Singapore, Taiwan, and Brunei Darussalam, while the Middle Eastern countries included were Saudi Arabia, Kuwait, the United Arab Emirates, Qatar, Bahrain, Oman, and Israel. The reasons of focusing on high‐income Asian and Middle Eastern countries were the rapid surge in EO‐CRC compared to low‐ and middle‐income Asian and Middle Eastern countries.^5^, ^6^, ^7^, ^8^, ^9^ To provide context for the findings, data from Western Europe and the United States were used as reference points for comparison. We followed the Global Burden of Disease (GBD) classification system for grouping of countries and territories based on their level of socio‐demographic development based on the World Bank data.^30^ The Global Burden of Disease (GBD) classification defines high‐income countries based on their Gross National Income (GNI) per capita. The World Bank's income classification is commonly used in conjunction with GBD studies to categorize countries. According to the World Bank's classification, high‐income countries are those with a high standard of living and strong economies. They typically have a diverse range of industries, well‐developed infrastructure, and advanced healthcare systems.^30^

### 2.4 Estimation of mortality‐to‐incidence ratio (MIR)

The colorectal cancer mortality‐to‐incidence ratio (MIR) was calculated for both genders by dividing the age‐specific mortality rate per 100,000 for colorectal cancer by the age‐specific incidence rate per 100,000 for each nation in the sample for a given year. The MIR serves as a broad comparative measure for assessing disparities in cancer outcomes. While MIR is less refined than relative survival, it enables a quick cross‐national contrast of survival rates as incidence and mortality data are widely accessible for most countries, making it a desirable and straightforward approach for international comparisons.^25^

### 2.5 Confounding factors

In order to account for variations in development indicators such as incomes per capita, average educational attainment, and fertility rates across countries, we utilized the socio‐demographic index (SDI) from 1990 to 2019 for all countries included in the study. The socio‐demographic index (SDI) is a composite indicator that incorporates three key indicators: lag‐distributed income per capita, average educational attainment, and the total fertility rate among individuals aged below 25 years. By combining these indicators, the SDI provides a comprehensive measure of the socio‐demographic development level in each country, taking into account economic factors, education levels, and fertility patterns.^31^ This helped to control the spectrum of development among the study populations. Additionally, to adjust for risk factors beyond education, we incorporated the summary exposure value (SEV) from 1990 to 2019 for all included countries. The SEV represents a risk‐weighted prevalence of a specific risk factor exposure, allowing us to account for a broader range of risk factors in our analysis.^31^

### 2.6 Statistical analysis

The study aimed to understand how the rates of an EO‐CRC changed between 1990 and 2019 and whether these changes were related to gender. To explore how the rates of an EO‐CRC changed overtime, the absolute differences in annual incidence rates between 1990 and 2019 were computed and examined for trends and comparisons. To evaluate the relationship between gender as an independent factor and the mortality‐to‐incidence ratio (MIR) as an outcome, generalized linear models were used. All models were adjusted for socio‐demographic index (SDI) and summary exposure value (SEV). We further included an interaction term for gender with year, to test whether the relationship between gender and MIR is changing over the 30 years. Ordinary least squares, including Student's *t* test, ANOVA, and linear regression, were employed for this purpose. Sixteen models were created using the MIR of different countries as outcomes with choosing the appropriate outcome distribution (the normal, Poisson, and gamma distributions). To check for normality, Q‐Q plots and kernel density plots were used. The homoskedasticity assumption (constant variance) was tested using the White test.^32^, ^33^ All *p* values were from two‐sided tests, and results were deemed statistically significant at *p* < 0.05. All analyses were performed using SAS statistical software version 9.4 (SAS Institute Inc.).

## 3 RESULTS

### 3.1 Male incidence, mortality crud rates, MIR

**FIGURE 1:**
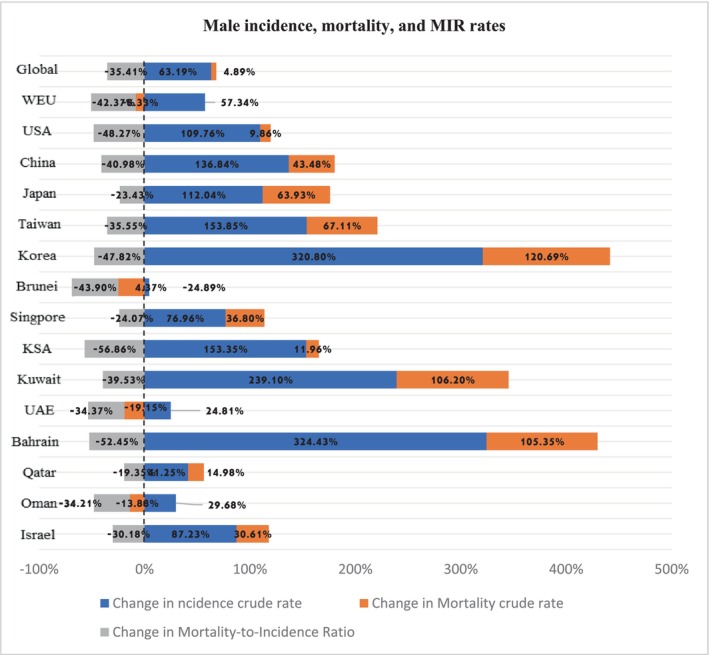
Male incidence, mortality, and MIR rates.

The incidence and mortality statistical data for male diagnosed with EO‐CRC from the worldwide, reference courtiers (Western Europe and the United States), and all included nations are shown in Table 1 and Figure 1. The EO‐CRC incidence rate for males had a varied increase from 1990 to 2019, except for the United States where there was a 41.25%; 95% confidence uncertainty (CU) (36.19–44.63) increase in incidence rate. China had the highest EO‐CRC incidence rate increase by 324%, followed by Saudi Arabia with 320%; 95% CU (283.87–351.06). The highest mortality crude rate increase was for Saudi Arabia 120.69%; 95% CU (119.44–130.59), followed by Taiwan 106.2%; 95% CU (102.11–114.91). Singapore had the lowest mortality crude rate with a decline of −24.89% over time. The highest MIR decrease was for Taiwan −56.86%; 95% CU (−56.75 to −61.52) decline from 1990 to 2019, followed by the China −52.42%; 95% CU (−52.29 to −56.75). The lowest MIR decrease was for the United States with −19%; 95% CU (−19.32 to −20.93) decrease.

**TABLE 1:** Male mortality‐to‐incidence ratio in early onset of colorectal cancer in Asian high‐income countries from 1990 to 2019 (age 15–49).

		Incidence crude populations' rate (95% UI)			Mortality crude populations' rate (95% UI)			Mortality‐to‐Incidence ratios (95% UI)		
Countries		1990	2019	Change (%)	1990	2019	Change (%)	1990	2019	Change (%)
	Global	3.68 (3.48 to 3.95)	6.89 (6.16 to 7.75)	87.23 (84.34 to 93.65)	1.96 (1.83 to 2.14)	2.56 (2.31 to 2.82)	30.61 (30.43 to 33.12)	0.53 (0.5 to 0.54)	0.37 (0.35 to 0.38)	−30.18 (−27.7 to −30.22)
	Western Europe	7.21 (6.98 to 7.47)	9.35 (7.96 to 10.9)	29.68 (29.05 to 32.11)	2.81 (2.74 to 2.88)	2.42 (2.27 to 2.53)	−13.88 (−12.74 to −13.93)	0.38 (0.33 to 0.39)	0.25 (0.21 to 0.29)	−34.21 (−30.89 to 37.01)
	United States	8.46 (8.21 to 8.71)	11.95 (9.64 to 16.67)	41.25 (36.19 to 44.63)	2.67 (2.59 to 2.74)	3.07 (2.95 to 3.21)	14.98 (14.92 to 16.21)	0.31 (0.26 to 0.33)	0.25 (0.22 to 0.28)	−19.35 (−19.32 to −20.93)
Southeast and East Asia										
	China	3.93 (3.24 to to 4.67)	16.68 (13.2 to 20.78)	324.43 (283.87 to 351.06)	2.43 (1.99 to 2.93)	4.99 (3.97 to 5.97)	105.35 (102.71 to 113.99)	0.61 (0.57 to 0.65)	0.29 (0.24 to 0.32)	−52.45 (−52.29 to −56.75)
	Japan	11.73 (11.21 to 12.24)	14.64 (11.57 to 18.34)	24.81 (24.1 to 26.84)	3.76 (3.68 to 3.84)	3.04 (2.82 to 3.12)	−19.15 (−18.95 to −19.28)	0.32 (0.30 to 0.34)	0.21 (0.19 to 0.24)	−34.37 (−34.33 to −37.19)
	Taiwan	8.44 (7.77 to 9.14)	28.62 (21.24 to 37.61)	239.1 (191.75 to 258.73)	3.71 (3.45 to 3.98)	7.65 (5.76 to 9.93)	106.2 (102.11 to 114.91)	0.43 (0.40 to 0.46)	0.26 (0.22 to 0.29)	−39.53 (−39.45.‐42.77)
	South Korea	4.03 (3.48 to 4.55)	10.21 (7.8 to 13.05)	153.35 (144.08 to 165.94)	2.09 (1.83 to 2.33)	2.34 (1.89 to 2.52)	11.96 (11.93 to 12.94)	0.51 (0.45 to 0.54)	0.22 (0.17 to 0.24)	−56.86 (−56.75 to −61.52)
	Brunei	6.25 (4.76 to 7.95)	11.06 (8.23 to 14.51)	76.96 (73.33 to 83.27)	3.37 (2.64 to 4.22)	4.61 (3.45 to 6.07)	36.8 (36.35 to 39.82)	0.54 (0.51 to 0.58)	0.41 (0.4 to 0.43)	−24.07 (−23.97 to −26.04)
	Singapore	7.1 (6.19 to 8.07)	7.41 (5.48 to 9.95)	4.37 (4.35 to 4.72)	2.29 (2.63 to 3.34)	1.72 (1.43 to 2.04)	−24.89 (−22.84 to −25.02)	0.41 (0.37 to 0.44)	0.23 (0.21 to 0.26)	−43.9 (−43.7 to −47.5)
West Asia (Middle East)	Saudi Arabia	1.25 (0.75 to 1.96)	5.26 (3.51 to 7.76)	320.8 (308.22 to 347.18)	0.87 (0.53 to 1.38)	1.92 (1.31 to 2.82)	120.69 (119.44 to 130.59)	0.69 (0.64 to 0.74)	0.36 (0.32 to 0.39)	−47.82 (−47.75 to −51.74)
	Kuwait	1.69 (1.37 to 2)	4.29 (3.14 to 5.68)	153.85 (149.94 to 166.48	0.76 (0.62 to 0.9)	1.27 (0.94 to to 1.67)	67.11 (66.78 to 72.61)	0.45 (0.42 to 0.51)	0.29 (0.24 to 0.34)	−35.55 (−35.44 to −38,46)
	United Arab Emirates	1.91 (1.01 to 3.06)	4.05 (2.5 to 6.02)	112.04 (109.69 to 121.23)	1.22 (0.65 to 1.96)	2 (1.25 to 2.98)	63.93 (63.44 to 69.17)	0.64 (0.62 to 0.66)	0.49 (0.45 to 0.52)	−23.43 (−23.32 to −25.35)
	Bahrain	1.9 (1.46 to 2.41)	4.5 (3.21 to 6.14)	136.84 (133.36 to 148.07)	1.15 (0.89 to 1.47)	1.65 (1.46 to 2.41)	43.48 (43.27 to 47.05)	0.61 (0.57 to 0.64)	0.36 (0.32 to 0.39)	−40.98 (40.89 to 44.34)
	Qatar	1.23 (0.83 to 1.82)	2.58 (1.75 to 3.65)	109.76 (108.31 to 118.77)	0.71 (0.4 to 1.08)	0.78 (0.54 to 1.11)	9.86 (9.85 to 10.66)	0.58 (0.53 to 0.64)	0.3 (0.28 to 0.33)	−48.27 (48.12 to −52.23)
	Oman	1.43 (0.85 to 2.18)	2.25 (1.52 to 3.61)	57.34 (56.88 to 62.04)	0.84 (0.5 to 1.29)	0.77 (0.52 to 1.23)	−8.33 (−8.31 to −9.01)	0.59 (0.54 to 0.63)	0.34 (0.31 to 0.39)	−42.37 (42.27 to −45.84)
	Israel	3.83 (3.28 to 4.29)	6.25 (4.5 to 8.38)	63.19 (61.69 to 68.37)	1.84 (1.59 to 2.08)	1.93 (1.63 to 2.88)	4.89 (4.88 to 5.29)	0.48 (0.43 to 0.52)	0.31 (0.27 to 0.35)	−35.41 (35.39–38.31)

*Note*: Numbers in parenthesis represent 95% uncertainty intervals (UIs).

### 3.2 Female incidence, mortality crud rates, and MIR

**FIGURE 2:**
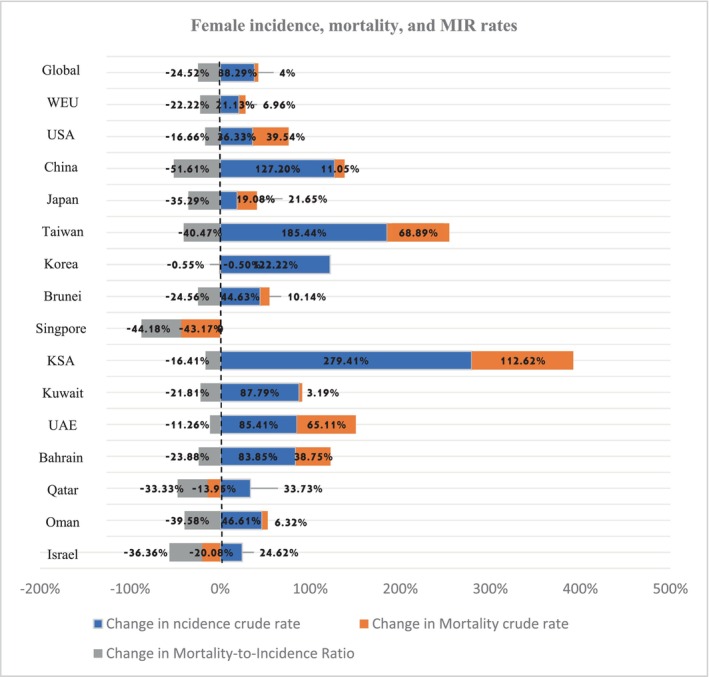
Female incidence, mortality, and MIR rates.

The incidence, mortality crude rates, and MIR data for female diagnosed with EO‐CRC form the global, and all included countries and regions are represented in Table 2 and Figure 2. The EO‐CRC incidence rate for females had a varied increase from 1990 to 2019, except for Singapore where there was no change in incidence rate. Saudi Arabia had the highest EO‐CRC incidence rate increase by 279.41%; 95% CU (271.61–302.34), followed by Taiwan with 185.44%; 95% CU (165.59–200.66). The highest mortality crude rate increase was for Saudi Arabia 112.62%; 95% CU (111.33–121.86), followed by Taiwan 68.89%; 95% CU (66.71–74.54). Singapore had the lowest mortality crude rate with a decline of −43.17% over time. The highest MIR decrease was for South Korea with a −55.55%; 95% CU (−55.52 to −56.52) followed by China with a −51.61%; 95% CU (−51.45 to −55.84) decline from 1990 to 2019. The lowest MIR decrease was the United Arab Emirates with a −11.26% 95% CU (−11.25 to −12.18) decline followed by Saudi Arabis with −16.41%; 95% CU (−16.33 to −17.75).

**TABLE 2:** Female mortality‐to‐incidence ratio in early onset of colorectal cancer in Asian high‐income countries from 1990 to 2019 (age 15 to 49).

		Incidence crude populations' rate (95% UI)			Mortality crude populations' rate (95% UI)			Mortality‐to‐Incidence ratios (95% UI)		
Countries		1990	2019	Change (%)	1990	2019	Change (%)	1990	2019	Change (%)
	Global	3.29 (3.07 to 3.53)	4.55 (4.11 to 5.01)	38.29 (35.14 to 38.76)	1.75 (1.61 to 1.91)	1.82 (1.66 to 1.99)	4 (3.98 to 4.32)	0.53 (0.49 to 0.57)	0.4 (0.36 to 0.43)	−24.52 (−24.48 to −26.53)
	Western Europe	6.67 (6.49 to 6.89)	8.08 (7.18 to 9.93)	21.13 (21.1 to 22.86)	2.44 (2.38 to 2.5)	2.02 (1.93 to 2.13)	6.96 (6.92 to 7.53)	0.36 (0.31 to 0.39)	0.28 (0.23 to 0.29)	−22.22 (−22.2 to −24.04)
	United States	7.21 (6.97 to 7.46)	9.83 (8.16 to 12.12)	36.33 (33.34 to 37.26)	2.2 (2.12 to 2.27)	3.07 (2.35 to 2.58)	39.54 (39.2 to 42.78)	0.3 (0.27 to 0.33)	0.25 (0.21 to 0.27)	−16.66 (−16.65 to −18.02)
Southeast and East Asia										
	China	3.21 (2.58 to 3.91)	7.31 (5.62 to 9.29)	127.2 (116.75 to 132.3)	1.99 (1.61 to 2.44)	2.21 (1.72 to 2.82)	11.05 (11.02 to 11.95)	0.62 (0.58 to 0.64)	0.3 (0.29 to 0.31)	−51.61 (−51.45 to −55.84)
	Japan	9.22 (8.21 to 9.61)	10.98 (8.44 to 13.96)	19.08 (17.51 to 19.35)	3.14 (3.07 to 3.21)	2.46 (2.32 to 2.57)	21.65 (21.5 to 23.42)	0.34 (0.31 to 0.37)	0.22 (0.2 to 0.24)	−35.29 (−35.24 to −38.18)
	Taiwan	5.91 (5.44 to 6.37)	16.87 (12.35 to 23.56)	185.44 (165.59 to 200.66)	2.53 (2.37 to 0.2.7)	4.29 (3.14 to 6.04)	68.89 (66.71 to 74.54)	0.42 (0.4 to 0.44)	0.25 (0.22 to 0.27)	−40.47 (−40.4 to −43.79)
	South Korea	3.68 (3.36 to 4.03)	8.18 (6.39 to 10.39)	122.22 (116.83 to 132.25)	2 (1.85 to 2.18)	1.99 (1.72 to 2.32)	−0.5 (−0.4 to −0.6)	0.54 (0.52 to 0.56)	0.24 (0.22 to 0.26)	−55.55 (−55.52 to −56.52)
	Brunei	5.87 (4.26 to 7.81)	8.49 (6.29 to 11.05)	44.63 (43.48 to 48.29)	3.35 (2.45 to 4.53)	3.69 (2.78 to 4.7)	10.14 (10.1 to 10.97)	0.57 (0.53 to 0.59_	0.43 (0.41 to 0.46)	−24.56 (−24.52 to −26.57)
	Singapore	6.21 (5.44 to 7.04)	6.21 (4.63 to 8.41)	–	2.71 (2.43 to 3.03)	1.54 (1.31 to 1.78)	−43.17 (−42.67 to −46.71)	0.43 (0.4 to 0.47)	0.24 (0.22 to 0.26)	−44.18 (−44.09 to −47.81)
Middle East	Saudi Arabia	1.02 (0.63 to 1.58)	3.87 (2.69 to 5.39)	279.41 (271.61 to 302,34)	1.03 (0.64 to 1.63)	2.19 (1.52 to 3.05)	112.62 (111.33 to 121.86)	1.008 (1.0 to 1.09)	0.56 (0.54 to 0.58)	−16.41 (−16.33 to −17.75)
	Kuwait	1.72 (1.41 to 2.06)	2.24 (1.61 to 2.98)	87.79 (87.35 to 94.99)	0.94 (0.78 to 1.13)	0.97 (0.7 to 1.32)	3.19 (3.18 to 3.45)	0.55 (0.52 to 0.59)	0.43 (0.4 to 0.47)	−21.81 (−21.78 to −23.6)
	United Arab Emirates	2.4 (1.48 to 3.91)	4.45 (2.87 to 6.6)	85.41 (83.7 to 92.42)	1.72 (1.05 to 2.81)	2.84 (1.82 to 4.17)	65.11 (64.4 to 70.45)	0.71 (0.67 to 0.74)	0.63 (0.6 to 0.67)	−11.26 (−11.25 to −12.18)
	Bahrain	1.92 (1.5 to 2.39)	3.53 (2.56 to 4.75)	83.85 (82.52 to 90.73)	1.29 (1 to 1.61)	1.79 (1.28 to 2.43)	38.75 (38.56 to 41.93)	0.67 (0.64 to 0.71)	0.51 (0.49 to 0.54)	−23.88 (−23.84 to −25.77)
	Qatar	2.49 (1.64 to 3.75)	3.33 (2.36 to 4.56)	33.73 (33.45 to 36.49)	1.66 (1.11 to 2.47)	1.48 (1.05 to 2.03)	−13.95 (−13.92 to −15.09)	0.66 (0.64 to 0.68)	0.44 (0.42 to 0.47)	−33.33 (−33.25 to −36.06)
	Oman	1.18 (0.96 to 1.78)	1.73 (1.16 to 2.48)	46.61 (46.33 to 50.43)	0.79 (0.46 to 1.21)	0.84 (0.56 to 1.21)	6.32 (6.3 to 6.83)	0.48 (0.45 to 0.52)	0.67 (0.65 to 0.69)	−39.58 (−39.5 to −42.82)
	Israel	5.32 (4.71 to 6)	6.63 (4.86 to 8.05)	24.62 (24.3 to 49.24)	2.34 (2.08 to 2.61)	1.87 (1.63 to 2.16)	−20.08 (−19.98 to −21.72)	0.44 (0.42 to 0.47)	0.28 (0.25 to 0.31)	−36.36 (−36.22 to −39.34)

*Note*: Numbers in parenthesis represent 95% uncertainty intervals (UIs).

### 3.3 Trends in MIR by gender in Asian and Middle Eastern countries

**FIGURE 3:**
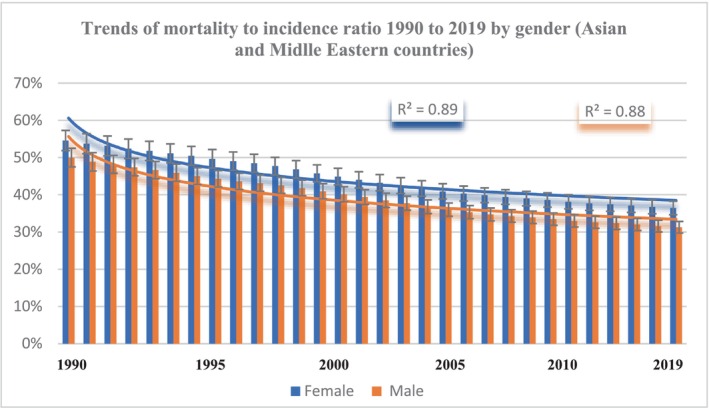
Trends of mortality‐to‐incidence ratio 1990 to 2019 by gender.

An overall decline in MIR was observed from 1990 to 2019 in Asian and Middle Eastern countries. Female MIR was consistently higher than male MIR, with male MIR decreasing from 51% in 1990 to 31% in 2019 and female MIR decreasing from 55% to 37% over the same period. The gap between male and female MIR increased over time in some Middle Eastern countries, with female MIR consistently higher than male MIR for the last 30 years. In 1990, female MIR was 4% higher than male MIR; in 2005, it was 5.26%; and in 2019, it was 6% (Figure 3).

### 3.4 Trends in MIR by gender in the Globe, the United States, and WE countries

**FIGURE 4:**
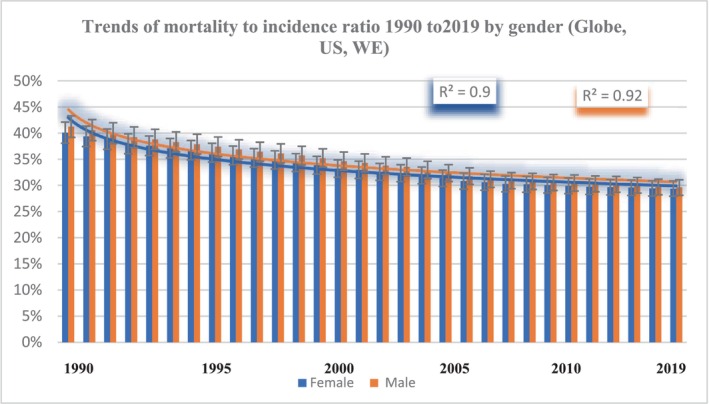
Trends of mortality‐to‐incidence ratio 1990 to 2019 by gender (Globe, the United States, WE).

There was a slight overall decline in MIR from 1990 to 2019 in the Globe, the United States, and WE countries. Male MIR was slightly higher than female MIR, with male MIR decreasing from 41.24% in 1990 to 29.59% in 2019 and female MIR decreasing from 40.1% to 29.37% over the same period. In 1990 (1.23%) and 2005 (1.18%), while in 2019, female MIR was slightly higher than male MIR (0.29%) (Figure 4).

### 3.5 The 30 years average combined MIR by gender from 1990 to 2019

**FIGURE 5:**
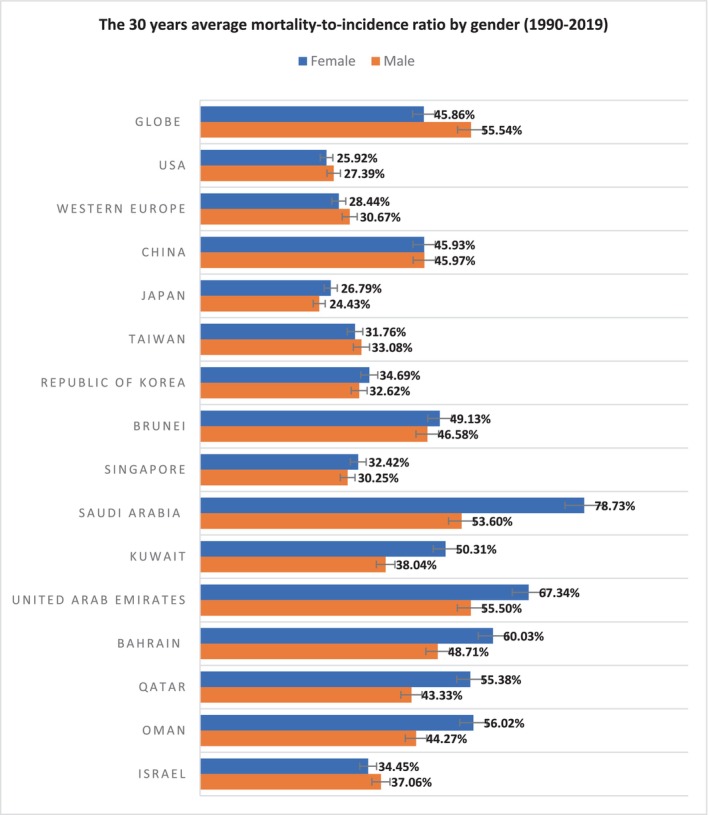
The 30 years average combined MIR by gender from 1990 to 2019.

Ten out of thirteen Asian and Middle Eastern countries combined had a higher MIR compared to their male counterparts. Among the studied countries, Saudi Arabia exhibited the highest MIR percentage for females, where 78.73% of the female EO‐CRC patients had died in a given year, followed by the United Arab Emirates with 67.34%. Similarly, the highest MIR percentage for males was observed in the United Arab Emirates with 55.5% of male EO‐CRC patients having died in a given year, followed by Saudi Arabia with 53.6% of female EO‐CRC patients having died in a given year. Notably, the MIR percentages for female patients in the globe, the United States, and WE were found to be relatively lower compared to their male counterparts during the studied period of 30 years (Figure 5).

### 3.6 The association between MIR and gender from 1990 to 2019 from regression model

**FIGURE 6:**
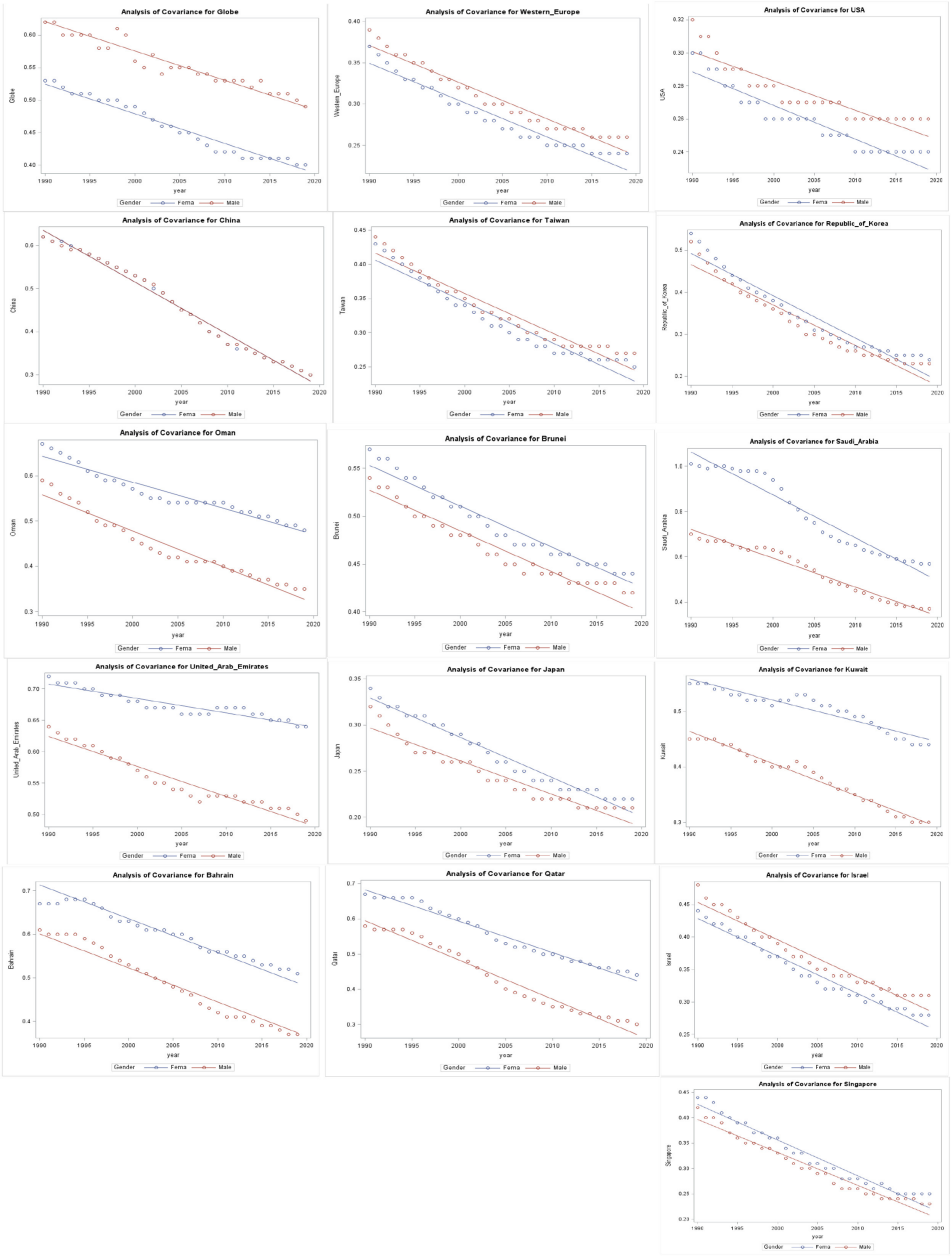
Association between MIR and gender from 1990 to 2019. The *p* values and the estimates from the generalized linear regression with an interaction term between ear and gender: Saudi Arabia *p* < 0.0001 *β*: 12.65, United Arab Emirates *p* < 0.0001 *β*: −4.84, Bahrain *p* < 0.0001 *β*: −0.007, Brunei *p* < 0.0001 *β*: −0.004, Kuwait *p* < 0.0001 *β*: −3.82, Oman *p* < 0.0001 *β*: −4.23, Qatar *p* < 0.0001 *β*: −4.44, Israel *p* 0.62 *β*: −0.3 (male is the reference group) The *p* values and the estimates from the generalized linear regression with an interaction term between ear and gender: Globe *p*: 0.008 *β*: 1.51, USA *p*: 0.53 *β*: 0.53, WE *p* < 0.0001 *β*: −0.004, China *p*: 0.73 *β*: 0.24, Japan *p*: 0.004 *β*: 1.43, South Korea *p*: 0.51 *β*: 0.88, Taiwan *p*: 0.59 *β*: 0.43, Singapore *p*: 0.13 *β*: 1.1 (male is the reference group).

The global male MIR was found to be significantly higher than that of female (*p*‐value 0.008, coefficient estimate: 1.51). In Asian countries, the differences in MIR between male and female were not significant, with the exception of Japan where female MIR was significantly higher than male (*p*‐value 0.004, coefficient estimate: 1.34). On the contrary, in Middle Eastern countries, Saudi Arabia had a significantly higher female MIR compared to their male counterparts (*p* < 0.0001, coefficient estimate: 12.65). Moreover, the study found that the gender gap in MIR has been increasing over time in most Gulf countries in the Middle East, except for Saudi Arabia where the difference has narrowed in the last 10 years. The survival outcome of female was significantly lower in Saudi Arabia and Japan (Figure 6).

## 4 DISCUSSION

The present study conducts a comprehensive analysis of the survival outcomes of EO‐CRC among high‐income Asian and Middle Eastern countries from 1990 to 2019 by utilizing the MIR, with a specific focus on examining the differences in gender. Despite the increasing incidence and mortality rates of EO‐CRC, the study reveals a decline in the MIR for both genders over the 30‐year period, indicating improved survival outcomes for EO‐CRC patients in these regions. However, it is noteworthy that males exhibited a greater improvement in survival outcomes compared to females across all selected countries, with a more significant reduction in male MIR over the past three decades. Furthermore, the study uncovers a consistent pattern of higher MIRs for females compared to males in Asian and Middle Eastern countries, with 10 out of 13 countries exhibiting higher MIRs for females throughout the study period.

In contrast to the global pattern, the United States and Western Europe (WE) displayed a reverse pattern, with higher MIRs observed in males over the 30‐year study period. Within the Middle Eastern countries, Saudi Arabia, followed by the United Arab Emirates, demonstrated significantly higher female MIRs than males, indicating a significant gender disparity in survival outcomes. Moreover, over the past decade, the gap between male and female MIRs in Middle Eastern countries, particularly in the Gulf States countries except Saudi Arabia, has widened. This suggests that gender disparities have persisted over time, with females experiencing poorer survival outcomes compared to males in these regions.

In Asian countries, there has been a notable improvement in survival outcomes for both genders. The reduction in the overall MIR among EO‐CRC patients in these high‐income Asian countries exceeds the global estimate and is comparable or even better outcomes than that of Western Europe and the United States. For instance, despite China experiencing the highest increase in EO‐CRC cases,^34^ the study reveals that China also had the highest decrease in MIR over the past 30 years. Similar patterns were observed in Singapore, Taiwan, and South Korea. These countries have made significant advancements in their healthcare systems in recent years, which may have contributed to the early detection and improved treatment of EO‐CRC cases.^35^, ^36^, ^37^ Moreover, they have implemented national screening programs for colorectal cancer for younger ages leading to early detection and better management of the disease.^38^ Furthermore, there has been a shift toward a healthier lifestyle in high‐income Asian countries, with an increase in physical activity and the adoption of healthier diets.^6^ These changes have resulted in a substantial reduction in obesity, a major risk factor for CRC, in these countries.^6^ This, in turn, may have contributed to a lower incidence of EO‐CRC cases and improved survival outcomes.

After accounting for differences in years, the study reveals that the MIR for females was significantly higher than that for males in Saudi Arabia and Japan populations. In comparison with Western Europe, the United States, and the other studied countries, Saudi Arabia exhibited the highest percentage of MIR for females, with an average of 78.73% of female EO‐CRC patients dying each year over the last 30 years. The emphasis on gender‐based survival differences appear to be most pronounced among Gulf States countries, particularly Saudi Arabia. These disparities in survival outcomes could be attributed to variations in exposure to risk factors, cancer care management, and screening practices across different countries.^39^, ^40^ The majority of EO‐CRC cases are believed to be sporadic, with various factors contributing to the development of sporadic colorectal cancer.^40^ Among the modifiable risk factors, physical inactivity, sedentary behavior, excessive caloric intake, and smoking have been identified as significant contributors.^41^, ^42^ These factors collectively lead to an energy imbalance, resulting in obesity, which is a significant risk factor for EO‐CRC, particularly in females.^43^ Therefore, the steady increase in EO‐CRC incidence in Gulf States countries may be attributed to the widespread adoption of inactive lifestyles and unhealthy eating habits.^44^, ^45^

Another significant contributing factor to the high incidence of EO‐CRC and low MIR in Gulf Countries may be attributed to the limited coverage and effectiveness of screening and prevention programs. While CRC screening programs have been implemented in Saudi Arabia and other Gulf countries, their efficacy remains limited.^46^ Recent studies have highlighted the urgent need to allocate more effort to improve screening program guidelines in order to address the substantial rise in EO‐CRC burden in Saudi Arabia and other Gulf countries.^47^, ^48^, ^49^ Moreover, the CRC screening recommendations, which are primarily based on age and gender, may need to be frequently re‐evaluated in light of emerging evidence on the epidemiology of EO‐CRC.

The findings underscore the importance of promoting CRC associated risk factors awareness, and screening in these regions to reduce the burden of EO‐CRC and improve survival outcomes for both genders. Cancer prevention strategies such as increasing public awareness of the risk factors for CRC and promoting healthy lifestyles should be prioritized. In addition, the promotion of healthy eating habits, regular physical activity, and tobacco cessation can also play a vital role in the young population at risk of CRC.^50^ The consistently higher MIR observed in females compared to males in Asian and Middle Eastern countries indicates that more attention should be paid to the unique challenges faced by female patients in the region. This could include increasing access to gender‐specific screening and diagnostic services, developing targeted prevention and treatment programs for women, and addressing cultural and social barriers that may prevent women from seeking timely medical care.^51^

There are a number of potential limitations to consider in this study. Firstly, the study only included high‐income Asian and Middle Eastern countries, which may not be representative of the overall populations in these regions including low‐ and middle‐income countries in Asia and Middle East. Secondly, the study focused on MIR as a measure of survival outcomes, which may not fully capture the complexity of the disease and its management. Thus, findings might vary with using actual survival rate. Finally, the study did not consider other potential confounding factors, such as access to health care, or comorbidities. Thus, the study is subject to confounding bias which may impact the results.

The present study highlights the significant improvement in survival outcomes for EO‐CRC patients in high‐income Asian countries. However, the study also reveals persistent gender disparities in survival outcomes, with females experiencing poorer outcomes compared to males in several areas of these regions particularly the Gulf States countries. The findings demonstrate the importance of investigating factors associated with female poor survival outcomes to reduce the burden of EO‐CRC and improve survival outcomes. Policymakers should focus on enhancing access to screening and early detection services for CRC, developing targeted prevention and treatment programs for women, and addressing cultural and social barriers that may prevent women from seeking timely medical care.

## DECLARATIONS

All authors confirm that they had full access to all the data in the study and accept responsibility to submit for publication.

## AUTHOR CONTRIBUTIONS

**Majed Ramadan:** Conceptualization (equal); data curation (equal); formal analysis (equal); investigation (equal); methodology (equal); software (equal); visualization (equal); writing – original draft (equal). **Rawiah A. Alsiary:** Project administration (equal); supervision (equal); validation (equal); writing – review and editing (equal). **Doaa A. Aboalola:** Project administration (equal); resources (equal); supervision (equal); validation (equal); writing – review and editing (equal).

## CONFLICT OF INTEREST STATEMENT

The authors declare that they have no conflict interests.

## ETHICS APPROVAL (IRB)

This study was performed in line with the principles of the Declaration of King Abdullah International Medical Research Center (KAIMRC). The Biomedical ethics committee in KAIMRC exempts this study from IRB due to public use data.

## Data Availability

The data that support the findings of this study are available from [Global Burden of Disease Study 2019]. No restrictions apply to the availability of these data, which were used under Public Use Files (PUF) data. Data are available at https://vizhub.healthdata.org.
